# Dietary Exposure to Mycotoxins through Alcoholic and Non-Alcoholic Beverages in Valencia, Spain

**DOI:** 10.3390/toxins13070438

**Published:** 2021-06-24

**Authors:** Dionisia Carballo, Mónica Fernández-Franzón, Emilia Ferrer, Noelia Pallarés, Houda Berrada

**Affiliations:** 1 Faculty of Agricultural Science, National University of Asunción, San Lorenzo 2160, Paraguay; dionisia.carballo@agr.una.py; 2 Laboratory of Food Chemistry and Toxicology, Faculty of Pharmacy, University of Valencia, 46100 Valencia, Spain; monica.fernandez@uv.es (M.F.-F.); Houda.berrada@uv.es (H.B.)

**Keywords:** mycotoxins, occurrence, beverages, risk assessment

## Abstract

The present study investigated the presence of 30 mycotoxins in 110 beverage samples of beer, wine, cava, and cider purchased in Valencia (Spain). A validated method based on dispersive liquid–liquid microextraction and chromatographic methods coupled with tandem mass spectrometry was applied. The method showed satisfactory recoveries ranging from 61 to 116% for the different beverages studied. The detection and quantification limits ranged from 0.03 to 2.34 µg/L and 0.1 to 7.81 µg/L, respectively. The results showed that beer samples were the most contaminated, even with concentrations ranging from 0.24 to 54.76 µg/L. A significant presence of alternariol was found in wine, which reached concentrations up to 26.86 µg/L. Patulin and ochratoxin A were the most frequently detected mycotoxins in cava and cider samples, with incidences of 40% and 26%, respectively. Ochratoxin A exceeded the maximum level set by the EU in one wine sample. The results obtained were statistically validated. The combined exposure was assessed by the sum of mycotoxin concentrations contaminating the same samples to provide information on the extent of dietary exposure to mycotoxins. No significant health risk to consumers was associated with the mycotoxin levels detected in the beverages tested.

## 1. Introduction

Mycotoxins are secondary metabolites produced by a wide variety of filamentous fungi, such as *Aspergillus*, *Fusarium*, *Penicillium* and *Alternaria*, which can grow under different climatic conditions on agricultural commodities. Pathogenic fungal toxins have been detected along the entire process of food production; in the field, during harvest, and during processing and storage, as well as in finished products [1,2]. Some mycotoxins have been associated with human and animal diseases; these are classified as carcinogens, hepatotoxins, nephrotoxins, or neurotoxins [3].

The consumption of alcoholic beverages is widespread; beer and wine are the most consumed beverages in the European Union [4]. Mycotoxins are commonly reported in fruits (grapes and other fruits), as well as in cereals (barley wheat and maize) used in wine and beer production [5,6].

European legislation has established maximum levels of ochratoxin A (OTA), recommending a tolerance level lower than 2.0 µg/L for all types of wine. However, there is no regulation for other mycotoxin levels in alcoholic beverages [7].

The occurrence of aflatoxins, ochratoxin, trichothecenes, alternaria toxins, and ergot alkaloids has already been investigated in some alcoholic beverages, such as wine [8,9] and beer [10,11,12,13].

Cava is a Spanish sparkling wine with a protected geographical status. It is made from several varieties of grape with a fermentation method similar to champagne. The process of obtaining cava by the traditional method involves two fermentation steps. During its first fermentation, must is converted into base wine while, during the second fermentation, sucrose, selected yeasts, and bentonite are added to the base wine, and the mixture is bottled and allowed to ferment and age in a cellar for 9 months for “cava” and 12 months for “champagne”. During this time, and after the second fermentation is complete, yeast autolysis takes place in the bottle. Finally, the yeasts are removed from the bottle by disgorging, and cava is marketed in the bottle that was used for aging [14,15]. Cider is a fermented beverage obtained from apple fruits. In Spain, cider is mainly produced in Asturias located on the Atlantic coast. Asturian cider apple varieties belonging to the protected designation of origin [16] were previously investigated by assessing their sugar, acid, and aroma contents [17].

Regarding mycotoxins in cider samples, scarce data are available; Tangni et al. [18] analyzed PAT in seven cider samples and Leblanc et al. [19] investigated twenty-one mycotoxins in two alcoholic beverage composites. However, no literature concerning mycotoxin levels in alcoholic beverages, such as cava, or beverage mixtures was found.

Dispersive liquid–liquid microextraction (DLLME) comprises a ternary component system formed by an aqueous solution, an organic extraction solvent (frequent solvent with high density), and a dispersive solvent (miscible in both of extractant and aqueous phases). This extraction method strongly depends on the adequate mixture of extraction and disperser solvents to reach the best conditions for efficient extraction. Moreover, DLLME offers some advantages such as high recovery and low-cost applications; it is also already applied in multimycotoxin analysis in several food samples [20,21,22,23].

Most mycotoxins analyses are carried out by liquid chromatography coupled with mass spectrometry; even gas chromatography is still preferred for the determination of trichothecenes. Both techniques enable the development of highly selective, sensitive, and accurate methods [24,25].

The aim of the present study was to evaluate the presence of thirty different mycotoxins, mainly *Alternaria* mycotoxins, trichothecenes, ochratoxin A, aflatoxins, patulin, zearalenone and its derivatives, fumonisins, and five emerging mycotoxins, in alcoholic and non-alcoholic beverages. The potential contribution of the studied beverages to mycotoxin dietary exposure has also been estimated.

## 2. Results and Discussion

### 2.1. Analytical Method Validation

Two MS/MS transitions acquired from each mycotoxin fragmented in a positive mode were used for mycotoxin quantification and confirmation. Recovery results were within the range of 61% and 116% intra- and inter-day data, respectively, ensuring repeatability and reproducibility (Table 1). Matrix effects (SSE) ranged from 71% to 114%, and matrix-matched calibration curves were used for quantification purposes. Limits of detection (LODs) and limits of quantification (LOQs) ranged between 0.03 and 2.34 µg/L and 0.1 and 7.81 µg/L, respectively.

### 2.2. Mycotoxin Occurrence in Beer Samples

**Figure 1:**
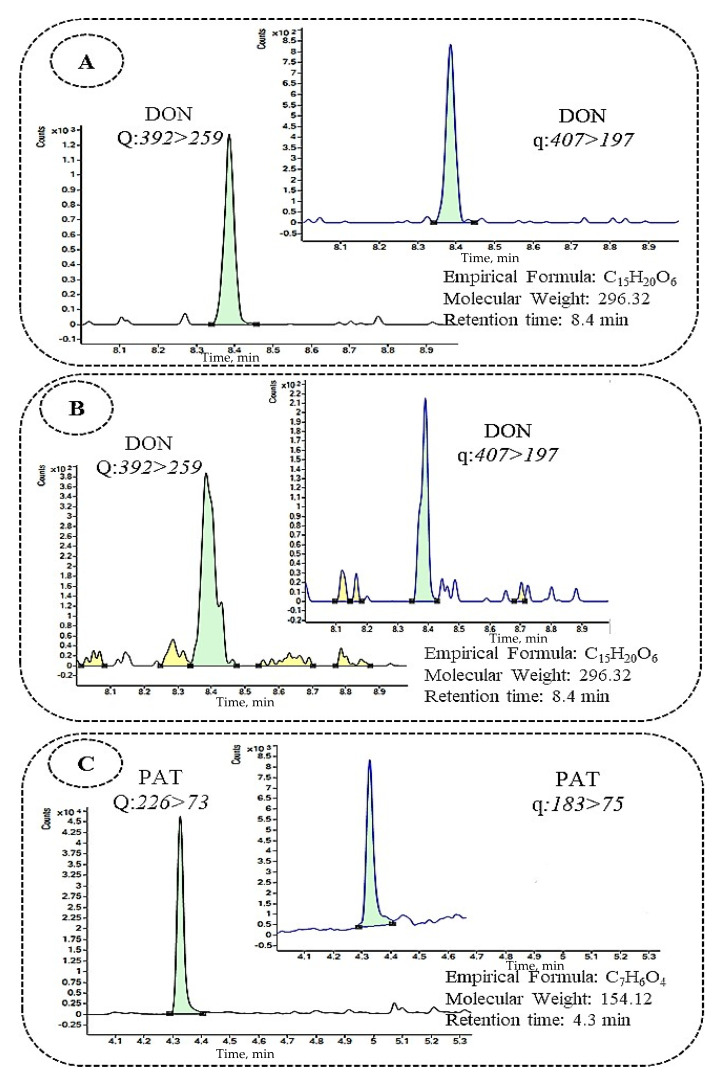
Chromatograms obtained from different beverage samples naturally contaminated with: (**A**) DON in beer sample (8.85 µg/L), (**B**) DON in wine sample (9.69 µg/L), and (**C**) PAT in cider sample (24.66 µg/L) through multiple reaction monitoring (MRM) by (GC-MS/MS).

All forty beer samples were found to be contaminated by at least one mycotoxin. AOH was the most prevalent mycotoxin in 90% of beer samples at mean levels of 19.39 µg/L, and the highest mean concentration was registered for PAT (43.18 µg/L), while the lowest incidence was for T2 toxin (29.88 µg/L) and the lowest mean concentration was observed for AFG1, with 1.16 µg/L (Table 2 and Figure 1). Bauer et al. [26] also detected AOH in 100% of the beer samples at 0.56 µg/L, and Prellé et al. [27] monitored AOH in 30% of beer samples at levels between 6.04 and 23.2 µg/L.

AOH and DON were the most frequently detected mycotoxins in A.F. beer, and the highest concentration reached was 43.19 µg/L for β-ZAL. The lowest incidence was detected for T-2 (10%), and the lowest mean concentration was 0.85 µg/L for AFG1.

Up to 87% of the beer samples from European markets have previously been reported as being contaminated with DON, at levels between 4 and 56.7 µg/L [28]. Other studies performed in Spain, Italy, and Estonia reported a slightly lower incidence in beer for DON (56% to 68%) and concentrations ranging from 2.1 to 73.2 µg/L [10,29,30].

In beer with lemonade, DON and OTA were the most prevalent mycotoxins, while the highest concentration was found for β-ZAL, with 42.97 µg/L. The lowest incidence was detected for ZON (10%) and the lowest contents for OTA with 1.83 µg/L. However, 15-ADON was only detected in A.F. beer samples, with an incidence of 40% at 12.08 µg/L. Juan et al. [29] also quantified 15-ADON in 6% of beer samples from Tunisia at similar mean levels.

NIV was present in 30% of beer samples at a mean concentration of 10.01 µg/L. Tamura et al. [31] also quantified NIV in 21% of beer samples from local supermarkets in Japan at a level under LOQ (˂5 ng/mL), while Bryla et al. [32] reported NIV in 39% of beer samples from different European producers at mean concentrations of 2.7 µg/L.

AFG1 and AFB1 were detected in 35% and 60% of beer and A.F. beer samples at mean levels of 1.16 to 1.88 µg/L, respectively. Burdaspal and Legarda [33] reported the presence of AFs in 64.3% of beer samples ranging from 0.07 to 4.94 ng/L. AFB1 was also detected in beer samples at low concentrations from 0.37 to 10.60 ng/L [34,35]. However, higher contents in beer, sometimes reaching concentrations of 35.5 µg/L, have already been reported [36,37].

ZON and β-ZAL were found in 8% and 25% of the A.F. and lemonade beer samples, at mean levels of 14.17 and 43.08 µg/L, respectively. Bauer et al. [26] also detected ZON in 100% of beer samples at a mean concentration of 0.96 µg/L.

OTA was detected in 80% of beer with lemonade at 1.83 µg/L. However, several studies reported a higher OTA incidence in beer samples. Coronel et al. [38] reported an OTA incidence of 89% in beer samples at a mean concentration of 0.02 µg/L in Catalonia (Spain); Czerwiecki et al. [39] reported OTA in 79% of beer samples in Poland, with a mean content of 25.7 µg/L; and Lasram et al. [40] reported OTA in 48% of domestic beer samples, with a mean content of 0.12 µg/L. However, low incidences were reported by Rubert et al. [41], who detected OTA in 10% of beer samples from Europe at a mean level of 3.2 µg/L.

Finally, PAT was detected in only 20% of AF beer at a mean level of 43.18 µg/L. Different technological processes applied in beer brewing, such as steeping, kilning, mashing, fermentation, and clarification, may influence its mycotoxin content [42]. A longer fermentation process could contribute to increased mycotoxin level transfer from cereal to malt and then to beer due to high thermal stability [10,30,42].

### 2.3. Mycotoxin Occurrence in Wine Samples

At least one mycotoxin was detected in 88% of wine samples. The most prevalent mycotoxin was AOH, with an incidence of 52%, while the highest contents were found for PAT and β-ZAL, with 24.64 and 25.86 µg/L, respectively. The lowest incidence was detected for β-ZAL and HT-2 toxins, and the lowest concentration was found for OTA, with 1.13 µg/L (Table 3)

The highest AME incidence was previously reported in wine samples (up to 93%) with mean values of up to 1.0 µg/L [43,44]. AOH was also reported in wine in more than 60% of samples, and at concentrations between 0.03 and 7.7 µg/L [43,44,45].

Concerning A.F. wine, OTA was the most frequently observed mycotoxin, even at a mean concentration of 1.08 µg/L. The lowest incidence was detected for PAT (30%), even at a mean level of 17.63 µg/L. Previous studies reported higher OTA incidence in wine ranging from 50% to 100% and concentrations of up to 8.6 µg/L [9,40,46]. In wine with lemonade, the highest incidences were for AOH and OTA up to 40%, while the highest concentration was found for βZAL with 25.86 µg/L.

Several studies have examined *Fusarium* mycotoxin monitoring in wine. Al-Taher et al. [47] identified T-2 in 11% of wine samples, with mean levels of 0.3 µg/L, and Logrieco et al. [5] reported the occurrence of FB2 in 17.6% wine commercialized in Italy, at levels ranging from 0.4 to 2.4 µg/L. In the present study, *Fusarium* mycotoxins are widely reported in common wine samples, where 15aDON, DON, HT-2, NEO, and NIV are detected, with incidences ranging from 10 to 60% and levels between 8.47 and 26.58 µg/L.

### 2.4. Mycotoxin Occurrence in Cava and Cider Samples

Despite the high prevalence of OTA in cava samples (80%), the concentrations detected were up to 1.36 µg/L; on the other hand, AOH showed up at 10%, even reaching 21.56 µg/L (Table 4). β-ZAL was the most detected mycotoxin in cider, reaching a mean concentration of 61.48 µg/L. PAT was found in 20% of samples, with a mean of 25.79 µg/L, and ZON was found at a level of 11.53 µg/L. In A.F. cider, PAT was the only mycotoxin detected, with an incidence of 30% and a mean concentration of 35.86 µg/L. Harris et al. [48] reported a PAT presence in 19% of USA cider samples at a mean concentration of 36.9 µg/L, and Leblanc et al. [19] quantified PAT in 50% of alcoholic beverage samples from French markets, including cider, at mean a level of 19.50 µg/L.

Some authors have reported that, although maceration could lead to an increase in mycotoxin production as consequence of long-term contact between grape skins and must, which itself favors the diffusion of mycotoxins from contaminated skins during alcoholic and malolactic fermentations, mycotoxigenic fungi growth is actually inhibited through this process. Furthermore, during fermentation, mycotoxins can also interact with yeast, lactic acid bacteria, or other compounds present, resulting in a decrease in mycotoxins [49].

Since most mycotoxins present in wines come from grapes, and cava is obtained from a wine base, information available in the literature about the presence of mycotoxins in wines is relevant as a means of analyzing mycotoxin contamination in cava. In this sense, Zwickel et al. [43] observed higher AOH incidences (93%) in red wine than those obtained in the present study in cava, even at a slightly lower contents of 7.7 µg/L. Regarding OTA in cava, its levels in this study were similar to those reported in a study by De Jesus et al. [46], which suggested an average concentration for total wine samples of 1.3 μg L.

**Figure 2:**
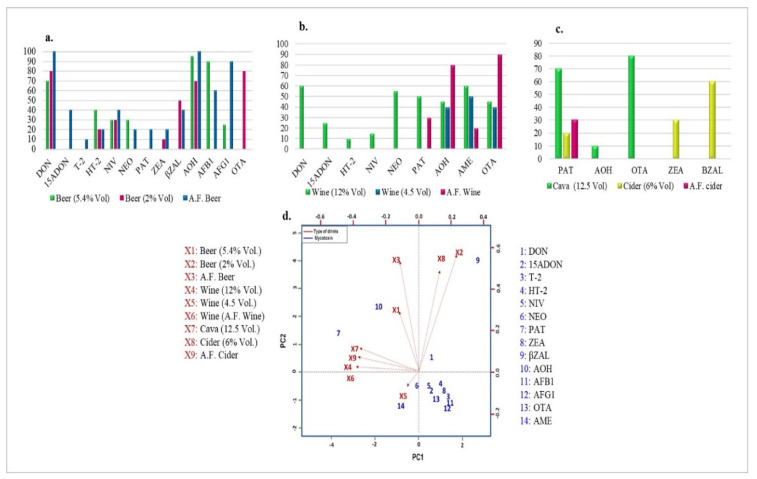
Incidence values (%) ((**a**) beer, (**b**) wine, (**c**) cava and cider) and biplot loading PCA ((**d**) PCA) of the different beverages.

To interpret the results in terms of incidence and contents, a principal component analysis (PCA) multivariate statistical analysis technique was used (Figure 2). The distribution map for the first principal components reached 35% for PAT in wine, cava, and A.F. cider, while the second component scored 26% for β-ZAL in beer with lemonade, A.F. beer, and cider. The results revealed similar behavior for these mycotoxins in the beverage groups mentioned. In A.F. beer samples, a similar trend was obtained for DON, NIV, and AOH. The same trend for AOH and OTA was shown in A.F wine. The highest incidence was observed for OTA and PAT in cava samples.

### 2.5. Multi-Mycotoxin Occurrence in the Analyzed Beverage Samples

Co-occurrence of mycotoxins in beer samples was found in 45% of beer samples, 20% of beer samples with lemonade, and 25% of A.F. beers. The sum of mycotoxin concentrations simultaneously contaminating the same samples ranged from 10.86 to 185.15 µg/L (Table 5). Rodriguez-Carrasco et al. [10] simultaneously detected DON and HT-2 in 9.1% of the analyzed samples. Similar results were reported by Juan et al. [29], who detected a co-occurrence of DON and 15ADON in 9% of the analyzed samples. Bertuzzi et al. [30] reported a co-occurrence of OTA-DON and OTA-FB1 in 41.5% and 42.4% of beer samples, respectively. Benesova et al. [35] found a co-occurrence of AFB2, AFG1, and AFG2 in 5.1% of samples at mean levels of 31 µg/L while, in a recent study in beer, Pascari et al. [11] reported a co-occurrence of DON, 3G-DON, and FB1.

Co-occurrence of mycotoxins in wine was found in 38% of wine samples, 5% of wine samples with lemonade, and 20% of A.F. wine. The sum of mycotoxin concentrations simultaneously present in the same positive samples reached 4.45 and 103.92 µg/L. Moreover, 60% of cava samples and 40% of cider samples were found to be concurrently contaminated with at least two mycotoxins, reaching concentrations from 17.60 to 25.52 µg/L and 46.86 to 129.37 µg/L, respectively.

### 2.6. Risk Assessment

The main contributors to the TDI of beer, A.F. beer, and beer with lemonade were HT-2 (9.82%), T-2 (3.64%), and β-ZAL (2.09%), respectively. The major contributors to TDI of wine, A.F. wine, and wine with lemonade were HT-2 (1.84%), OTA (0.31%), and β-ZAL (0.50%), respectively. Moreover, for cava samples, the main contributor was OTA, with 0.18%. Finally, in cider and A.F. cider samples, the main contributors to TDI were β-ZAL (0.24%) and PAT (0.08%), respectively (Table 6).

Regarding multicontaminated samples, an approximation of exposure assessment was also carried out. In terms of the sum of probable daily intake (PDI) values, a beer with lemonade could supply up to 8.90 ng/kg bw/day, while regular beer could contribute up to 47.54 ng/kg bw/day and A.F. beer could supply up to 21.16 ng/kg bw/day. In wine, the sum of PDI values ranged from 0.19 ng/kg bw/day for A.F. wine to 11.87 ng/kg bw/day for common wine intake. For cava, accumulative PDI values ranged from 0.25 to 0.36 ng/kg bw/day, while the sum of PDI values through cider consumption ranged from 0.40 to 1.1 ng/kg/bw/day (Table 5).

In fact, the values obtained in this study demonstrate that the intake of these mycotoxins by beverages consumption did not represent a toxicological concern, with exposure being far below the TDIs established by the Joint FAO/WHO Expert Committee on Food Additives (JECFA) [50].

## 3. Conclusions

In this survey, alcoholic and non-alcoholic beverages were evaluated for the presence of thirty mycotoxins. A total of 85% of the samples were contaminated with at least one mycotoxin. DON and AOH showed the highest incidences, followed by AME, OTA, and PAT. Per type of beverage, AOH and DON were the most frequently detected in beer, with 90% and 80% of the samples positive, respectively. AOH was even detected at high concentrations, up to 49.82 µg/L. A significant presence of AOH was also found in wine samples, and AOH was detected in 52% of samples at concentrations of up to 26.86 µg/L. In contrast, in cava and cider, OTA and PAT were detected the most, and they were present in 26% and 40% of samples, respectively. A high multi-occurrence of mycotoxins in different beverage samples was also observed, mainly in beer samples, while OTA was found in one wine sample, exceeding the maximum level established by the EU. However, the risk assessment carried out did not raise any toxicological concerns for consumers.

## 4. Materials and Methods

### 4.1. Chemicals and Reagents

Solvents (acetonitrile, hexane, ethyl acetate, chloroform, and methanol) were supplied by Merck (Darmstadt, Germany). Deionized water (<18.2 MΩ cm resistivity) was obtained in the laboratory using a Milli-QSP^®^ Reagent Water System (Millipore, Beadford, MA, USA). Ammonium formate (99%), formic acid (≥98%), and sodium chloride were supplied by Sigma Aldrich (Madrid, Spain). Syringe nylon filters (13 mm diameter and 0.22 µm pore size) were obtained from Analysis Vínicos S.L. The derivatization reagent composed of BSA (N,O-bis(trimethylsilyl) + TMCS (trimethylcholorosilane) + TMSI (N-trimethylsilyimidazole) (3:2:3) was obtained from Supelco (Bellefonte, PA, USA). Sodium dihydrogen phosphate and disodium phosphate, used to prepare phosphate buffer, were acquired from Panreac Química S.L.U. (Barcelona, Spain).

### 4.2. Standards and Solutions

Mycotoxin standards were purchased from Sigma Aldrich. Individual stocks of all analytes were prepared to obtain 20 mg/L in methanol and multianalyte working solutions. The multianalyte working standard solution of 2 mg/L was used for standard calibration curves, matrix-matched calibration curves, and recovery assays. All standards were stored in darkness and kept at −20 °C.

### 4.3. Procedures

#### 4.3.1. Samples

A total of 110 samples of beer (*n* = 40), wine (*n* = 40), cava (*n* = 10), and cider (*n* = 20) were purchased from different food stores located in Valencia from September 2017 to October 2018. Beer samples were divided into beer with an alcohol content up to 5.4% vol (*n* = 20), beer with lemonade (*n* = 10) containing 2% vol, and alcohol-free (A.F.) beer with an alcohol content of <1% vol (*n* = 10). Wine samples were classified into wine with an alcohol content of 12% vol (*n* = 20), wine with lemonade with an alcohol content up to 4.5% (*n* = 10), and A.F wine with an alcohol content of <1% vol (*n* = 10). Cider samples were separated in A.F. cider with an alcohol content of <1 % vol (*n* = 10) and normal cider (*n* = 10), while all cava samples contained a 12% alcohol vol (*n* = 10).

#### 4.3.2. Dispersive Liquid–Liquid Microextraction

Sample extraction was performed according to the method previously validated for tea beverages [51]. Briefly, prior to extraction, each bottle of beer, cava, and cider was gently shaken. Then, 100 mL was degassed by sonication for 15 min. Next, an aliquot of 5 mL was placed in a 10 mL conical tube, a mixture (950 µL of ACN) of dispersion solvent and (620 µL of EtOAc) of the extraction solvent was added, and the resulting mixture was shaken for 1 min. The mixture was centrifuged at 4000 rpm for 5 min, and the organic phase at the top of the tube was placed in a second conical tube. Next, a mixture of dispersion solvent (950 µL of MeOH) and extraction solvent (620 µL of CHCl3) was added to the remaining residue and, after agitation and centrifugation, the separated organic phase was added to the first organic phase. The solvent in the conical tube containing the two recovered phases was evaporated to near dryness under a nitrogen stream using a turbovap LV Evaporator (Zimark, Hopkinton, MA, USA). The dry residue was reconstituted with 1 mL of 20 mM ammonium formate (MeOH/ACN) (50/50 *v*/*v*) and filtrated.

### 4.4. GC–MS/MS Analysis

Gas Chromatographic analysis was carried out using an Agilent 7890A GC system coupled with an Agilent 7000A triple quadruple mass spectrometer with inter electron-impact ion source (EI, 70Ev). Quantitative data were acquired at selection reaction monitoring mode. The transfer line and source temperatures were 280 °C and 230 °C, respectively.

Analytes were separated on a HP-5MS 30 m × 0.25 mm × 0.25 µm capillary column. One microliter of the final mycotoxin-cleaned extract s was injected in splitless mode into the programmable temperature vaporization (PTV) inlet at 250 °C, using helium as carrier gas at a fixed pressure of 20.3 psi. The dry extract was then derivatized. Details of the procedure were described in a previously published study [10].

For quantification of each analyte, two selected reaction monitoring (SRM) transitions were required. The most intense SRM transition was selected for quantification purposes, as outlined in the requirements for mass spectrometry [52] (Table 1).

### 4.5. LC–MS/MS Analysis

HPLC-MS/MS analysis was performed using an Agilent 1200 liquid chromatography (Agilent Technologies, Palo Alto, CA, USA) coupled with a 3200 QTRAP^®^ ABSCIEX (Applied Biosystems, Foster City, CA, USA) equipped with a Turbo-VTM source (ESI) interface. The chromatographic separation of the analytes was performed at 25 °C with a reverse analytical column Gemini^®^ NX-C18 (3 µM, 150 × 2 mm ID) and guard column C18 (4 × 2 mm, ID; 3 µM). Mobile phases were a time-programmed gradient using water as phase A (5 mM ammonium formate and 0.1% formic acid) and methanol as phase B (5 mM ammonium formate and 0.1% formic acid). The gradient program started with a proportion of 0% for eluent B, increased to 100% in 10 min, decreased to 80% in 5 min, and finally to 70% in 2 min. Over the next 6 min, the column was cleaned, readjusted to initial conditions, and equilibrated for 7 min.

### 4.6. Method Validation

The analytical method was validated in-house according to the criteria established in SANTE 11813/2017 Document [52] with respect to the main analytical parameters of linearity, recovery, LODs, LOQs, and matrix effect. Due to their similar elaboration and fermentation processes, cava and cider were grouped with wine beverages, as conducted in a previous study by Ruíz-Delgado et al. [53].

Both external calibration curves and matrix-matched calibration curves were performed in triplicate, at concentrations of 0.1, 0.5, 1, 5, 10, 50, 100, and 250 µg/L, and linearity was expressed by the square correlation coefficient (r2). Precision was calculated in terms of the relative standard deviation (RSD). For the evaluation of matrix effects, signal suppression/enhancement (SSE) was compared based on the slopes of calibration curves (A/B*100), where “A” corresponded to the area of the matrix-matched standard and “B” corresponded to the area of the standard solution. SSE values higher than 100% indicated enhancement of the signal while those lower than 100% indicated the opposite. The accuracy of the method was evaluated by measuring the recoveries from blank samples spiked at 50, 100, and 200 µg/L. Precision studies were determined in fortified beer and wine, including similar beverages at the same levels, as previously mentioned, and were calculated as relative standard deviation percent (RSD%). Both recovery and precision studies were performed in triplicate on the same day (intra-day precision) and on three different days (inter-day precision) by prepared analysis (*n* = 9) at three spiked levels. Limits of detection (LODs) and quantification (LOQs) were determined as the concentrations for which respective signal-to-noise ratios (S/N) of ≥3 and ≥10 were validated from chromatograms of samples spiked at the lowest level.

### 4.7. Statistical Analysis

Principal component analysis (PCA) was performed using the mixOmics based on Omics Data Integration Project and R package version 6.1.1 [54]. A factor analysis was established to evaluate possible associations between studied analytes and beverage groups.

### 4.8. Mycotoxin Dietary Intake Calculation

A deterministic approach was performed for risk assessment. The exposure was estimated by the probable daily intake (PDI) which combined the average amount of mycotoxins found in the different analyzed samples with the beverage consumption estimation in the Spanish adult population. According to the Spanish Ministry of Agriculture and Environment, the annual consumption of different beverages was as following: beer (15.39 L), non-alcoholic beer (3.11 L), wine (3.02 L), other beverages mixed with wine (1.25 L), cava (0.58 L), and cider (0.25 L) [55]. The PDI [µg L^−1^ per body weight (bw)/day] of each mycotoxin was calculated as shown in the following equation [56]:

PDI = (C*K)/bw(1)

where “C” is the average concentration of mycotoxin detected in a beverage expressed as µg/L, “K” represents different beverage consumption expressed in L per day, and “bw” is the average weight used for the adult population (estimated at 70 kg). The health risk characterization of mycotoxin (% of relevant TDI) was performed by comparing the PDI with a tolerable daily intake (TDI) (µg/L bw day) of the following equation:

%TDI = (PDI/TDI)*100(2)

In order to evaluate consumers’ exposure to multicontaminated samples, an approximation of exposure assessment was also carried out. For this, the concentrations of the mycotoxins found in a multi-contaminated sample were obtained by determining the contamination range; thus, Ʃ*Cmin* and Ʃ*Cmax* were generated for the analyzed samples. Then, a combined health risk characterization $\left(\overset{i}{\underset{n=1}{\sum }}\%TDI\right)$ was proposed as follows:

(3) $$\sum_{n=1}^{i}\%TDI\;min=\sum_{n=1}^{i}\left(Cmin*K\right)/TDI$$

(4) $$\sum_{n=1}^{i}\%TDI\;max=\sum_{n=1}^{i}\left(Cmax*K\right)/TDI$$

According to the safety guidelines of the Joint FAO/WHO Expert Committee on Food Additives and the Scientific Committee on Food, TDIs in ng/kg bw were established as the following: 250 for ZON, 100 for the sum of T-2 and HT-2, 1200 for NIV, 1000 for DON and their acetyl forms (as 3-ADON and 15-ADON), and 400 for PAT. For OTA, a tolerable weekly intake of 120 ng/kg bw was established [50,57]. Aflatoxins are carcinogenic and their intake should be reduced to as low as is reasonably achievable.

## Figures and Tables

**Table 1:** Mass spectrometry transitions, limits of detection and quantification (LODs, LOQs), matrix effects (SSE%), recovery at different spiked concentrations using the chromatographic methods of tandem mass spectrometry.

Mycotoxin ^g^	RT ^a^ (min)	Transitions		Beer						Wine and Similar Beverages					
		Quantitative	Qualitative				Recovery (%)						Recovery (%)		
				LOD ^b^	LOQ ^c^	SSE ^d^	Spiked Level µg/L			LOD ^b^	LOQ ^c^	SSE ^d^	Spiked Level µg/L		
				µg/L	µg/L	(%)	50	100	200	µg/L	µg/L	(%)	50	100	200
DON ^e^	8.4	392 > 259	407 > 197	0.58	1.95	95	68	69	71	0.58	1.95	96	79	77	71
3-ADON ^e^	9.45	392 > 259	467 > 147	1.17	3.90	78	69	100	106	1.17	3.90	94	100	99	104
15-ADON ^e^	9.65	292 > 217	392 > 184	0.58	1.95	92	87	97	91	0.58	1.95	92	103	101	102
DAS ^e^	9.73	350 > 229	378 > 124	0.58	1.95	96	67	78	99	0.58	1.95	96	116	94	98
NEO ^e^	11.68	252 > 195	252 > 167	0.58	1.95	92	67	93	83	0.58	1.95	96	96	98	101
NIV ^e^	10.15	289 > 73	379 > 73	2.34	7.81	94	71	75	88	2.34	7.81	94	114	114	101
T-2 ^e^	14.39	350 > 244	350 > 229	2.34	7.81	72	69	91	102	2.34	7.81	74	107	100	99
HT-2 ^e^	14.80	347 > 157	347 > 185	0.58	1.95	78	71	113	107	1.17	3.90	94	86	101	101
PAT ^e^	4.3	226 > 73	183 > 75	2.34	7.81	81	74	81	92	1.17	3.90	84	61	96	91
FUS-X ^e^	9.55	450 > 260	450 > 245	2.34	7.81	93	89	84	80	1.17	3.90	100	87	97	96
ZON ^e^	15.95	462 > 151	462 > 333	2.34	7.81	111	67	77	97	1.17	3.90	90	108	103	91
α-ZAL ^e^	15.45	433 > 309	433 > 295	1.17	3.90	101	89	99	107	0.58	1.95	114	98	99	109
β-ZAL ^e^	15.68	307 > 292	307 > 277	2.34	7.81	72	72	67	106	2.34	7.81	101	95	66	87
α-ZOL ^e^	16.45	305 > 289	305 > 73	1.17	3.90	87	75	71	93	2.34	7.81	104	77	100	96
β-ZOL ^e^	16.83	536 > 446	536 > 333	1.17	3.90	93	66	73	108	2.34	7.81	71	106	104	106
AFB_1_^f^	7.41	313 > 241	313 > 289	0.06	0.2	85	83	86	81	0.3	1	79	71	79	98
AFB_2_^f^	7.36	315 > 286	315 > 259	0.3	1	95	85	97	85	1.5	5	81	78	89	83
AFG_1_^f^	7.23	329 > 243	329 > 311	0.06	0.2	77	81	92	82	0.3	1	91	111	98	70
AFG_2_^f^	7.13	331 > 313	331 > 245	0.3	1	81	70	108	101	1.5	5	79	86	85	109
AOH ^f^	8.03	259 > 128	259 > 184	0.3	1	111	89	111	91	0.03	0.1	92	101	90	107
AME ^f^	9.10	273 > 128	273 > 228	1.5	5	78	76	98	103	0.3	1	84	99	76	89
FB_1_^f^	7.7	722 > 334	722 > 352	1.5	5	71	83	71	87	1.5	5	76	69	71	79
FB_2_^f^	7.85	706 > 336	706 > 318	1.5	5	87	76	69	82	1.5	5	86	71	65	69
ENN A ^f^	11.74	699 > 228	699 > 210	0.03	0.1	110	69	85	82	0.15	0.5	71	72	85	87
ENN A_1_^f^	11.3	685 > 214	685 > 210	0.15	0.5	106	85	94	93	0.03	0.1	86	69	82	102
ENN B ^f^	10.73	657 > 196	657 > 214	0.15	0.5	92	91	103	98	0.15	0.5	111	73	85	86
ENN B_1_^f^	10.68	671 > 214	671 > 228	0.03	0.1	91	72	114	102	0.15	0.5	88	73	85	86
BEA ^f^	10.84	801 > 784	801 > 244	0.3	1	75	98	94	96	1.5	5	87	75	83	88
STG ^f^	9.08	325 > 281	325 > 310	1.5	5	96	83	93	81	1.5	5	95	85	81	89
OTA ^f^	8.68	404 > 102	404 > 239	0.06	0.2	99	79	89	87	0.15	0.5	104	107	84	85

^a^ RT = retention time; ^b^ LOD = limit of detection; ^c^ LOQ = limit of quantification; ^d^ SSE = signal suppression/enhancement; ^e^ GC-MS/MS determination; ^f^ LC-MS/MS determination; ^g^ DON = deoxynivalenol; 3-ADON = 3-acetyl-deoxynivalenol; 15-ADON = 3-acetyl-deoxynivalenol; DAS = diacetoxyscirpenol; NEO = neosolaniol; NIV = nivalenol; T-2 and HT-2 toxins; PAT = patulin; FUS-X = fusarenon-X; ZON = zearalenone; α-ZAL = α-zearalanol; β-ZAL = β-zearalanol; α-ZOL = α-zearalenol; β-ZAL = β-zearalenol; AFB_1_, AFB_2_, AFG_1_, AFG_2_ = four aflatoxins B_1_, B_2_, G_1_, G_2_; AOH = alternariol; AME = alternariol-methyl- ether; FB_1_, FB_2_ = fumonisins B_1_, B_2_, ENN A, ENN A_1_; ENN B ENN B_1_ = enniatins A, A_1_, B, B_1_; BEA = beauvericin; STG = sterigmatocystin; OTA = ochratoxin A.

**Table 2:** Incidence, mean concentration, and determined mycotoxins range in beer samples.

Mycotoxin	Beer ^a^ (*n* = 20)			A.F. Beer ^b^ (*n* = 10)			Beer with Lemonade (*n* = 10)			TOTAL (*n* = 40)		
	I ^e^ (%)	Mean µg/L	Range µg/L	I ^e^ (%)	Mean µg/L	Range µg/L	I ^e^ (%)	Mean µg/L	Range µg/L	I ^e^ (%)	Mean µg/L	Range µg L
AFB1 ^c^	90	1.06 ± 0.15	0.87–1.38	60	2.70 ± 4	1–10.60	-	n.d.	n.d.	60	1.88 ± 1	0.87–10.60
AFG1 ^c^	25	1.47 ± 0.9	0.43–2.92	90	0.85 ± 0.6	0.7–1.98	-	n.d.	n.d.	35	1.16 ± 1	0.43–2.92
AOH ^c^	95	24.93 ± 10.42	8.83–49.82	100	28.81 ± 9	20.25–48.37	70	4.44 ± 2	2.01–8.32	90	19.39 ± 13	2.01–49.82
15-ADON ^d^	-	n.d.	n.d.	40	12.08 ± 1	10.78–12.93	-	n.d.	n.d.	10	12.08 ± 1	10.78–12.93
β-ZAL ^d^	-	n.d.	n.d.	50	43.19 ± 9	31.46–54.76	50	42.97 ± 1.89	40.43–45.25	25	43.08	31.46–54.76
DON ^d^	70	8.65 ± 0.12	8.50–8.82	100	9.63 ± 1	8.58–11.82	80	8.76 ± 0.28	8.44–9.35	80	9.01 ± 0.5	8.44–11.82
HT-2 ^d^	40	16.31 ± 1	14.42–18.59	20	14.43 ± 5	11.20–17.67	20	15.15 ± 0.07	15.10–15.21	30	15.29 ± 0.9	11.20–18.59
NEO ^d^	30	14.20 ± 0.25	13.86–14.46	20	15.17 ± 2	13.90–16.44	-	n.d.	n.d.	20	14.67 ± 0.6	13.86–16.44
NIV ^d^	30	10.40 ± 1	8.96–14.01	40	10.34 ± 2	8.96–12.52	30	9.34 ± 0.24	9.13–9.60	30	10.01 ± 0.5	8.96–14.01
OTA ^c^	-	n.d.	n.d.	-	n.d.	n.d.	80	1.83 ± 1.18	0.24–3.38	20	1.83 ± 1	0.24–3.38
PAT ^d^	-	n.d.	n.d.	20	43.18 ± 0.4	42.89–43.48	-	n.d.	n.d.	5	43.18 ± 0.4	42.89–43.48
T-2 ^d^	-	n.d.	n.d.	10	29.88 ± 8	29.88 ± 8	-	n.d.	n.d.	3	29.88 ± 8	29.88 ± 8
ZON ^d^	-	n.d.	n.d.	20	14.95 ± 1	13.80–16.10	10	13.60 ± 0.2	13.60 ± 0.2	8	14.17 ± 0.9	13.60–16.10

^a^ Beer = beer with alcohol; ^b^ A.F. beer = alcohol free beer; ^c^ mycotoxin determined by LC-MS/MS; ^d^ mycotoxin determined by GC-MS/MS; ^e^ incidence (%): (number positive samples/number total samples) × 100.

**Table 3:** Incidence, mean concentration, and determined mycotoxin range in wine samples.

Mycotoxin	Wine ^a^ (*n* = 20)			A.F. Wine ^b^ (*n* = 10)			Wine with Lemonade (*n* = 10)			TOTAL (*n* = 40)		
	I ^e^ (%)	Mean µg/L	Range µg/L	I ^e^ (%)	Mean µg/L	Range µg/L	I ^e^ (%)	Mean µg/L	Range µg/L	I ^e^ (%)	Mean µg/L	Range µg/L
AOH ^c^	45	7.79 ± 8	1.55–26.86	80	5.35 ± 3	0.83–9.29	40	2.56 ± 2	0.61–4.65	52	5.23 ± 2	0.61–26.86
AME ^c^	60	7.55 ± 5	1.36–18.05	50	16.40 ± 4	11.14–23.13	20	12.33 ± 2	10.82–13.85	50	12.09 ± 4	1.36–23.13
15-ADON ^d^	25	11.28 ± 0.5	10.61–11.91	-	n.d.	n.d.	-	n.d.	n.d.	12	11.28 ± 0.5	10.61–11.91
β-ZAL ^d^	-	n.d.	n.d.	-	n.d.	n.d.	20	25.86 ± 3	23.33–28.40	5	25.86 ± 3	23.33–28.40
DON ^d^	60	8.85 ± 0.3	8.47–9.69	-	n.d.	n.d.	-	n.d.	n.d.	30	8.85 ± 0.3	8.48–9.69
HT-2 ^d^	10	15.65 ± 0.1	15.55–15.75	-	n.d.	n.d.	-	n.d.	n.d.	5	15.65 ± 0.1	15.55–15.75
NEO ^d^	45	14.27 ± 0.3	13.89–14.97	-	n.d.	n.d.	-	n.d.	n.d.	22	14.27 ± 0.3	13.89–14.97
NIV ^d^	15	21.26 ± 4	18.06–26.58	-	n.d	n.d.	20	10.55 ± 0.6	10.07–11.03	12	16.05 ± 7	10.07–26.58
OTA ^c^	45	1.12 ± 0.5	0.66–2.28	90	1.08 ± 0.2	0.57–1.50	40	1.21 ± 0.4	0.60–1.79	47	1.13 ± 0.06	0.57–2.28
PAT ^c^	50	31.66 ± 22	15.35–88.24	30	17.63 ± 4	14.67–22.97	-	n.d.	n.d.	32	24.64 ± 9	15.35–88.24

^a^ Wine = with alcohol; ^b^ A.F. wine = alcohol free wine; ^c^ mycotoxin determined by LC-MS/MS; ^d^ mycotoxin determined by GC-MS/MS; ^e^ incidence (%): (number positive samples/number total samples) × 100.

**Table 4:** Incidence, mean concentration, and determined mycotoxin range in cava and cider samples.

Mycotoxin	Cava (*n* = 10)			Cider ^a^ (*n* = 10)			A.F. Cider ^b^ (*n* = 10)			TOTAL (*n* = 30)		
	I ^e^ (%)	Mean µg/L	Range µg/L	I ^e^ (%)	Mean µg/L	Range µg/L	I ^e^ (%)	Mean µg/L	Range µg/L	I ^e^ (%)	Mean µg/L	Range µg/L
AOH ^c^	10	21.56 ± 1	21.56 ± 1	-	n.d	n.d.	-	n.d.	n.d.	3	21.56 ± 1	21.56 ± 1
β-ZAL ^d^	-	n.d.	-	60	61.48 ± 30	25.17–102.96	-	n.d.	n.d.	20	61.48 ± 30	25.17–102.96
OTA ^c^	80	1.36 ± 0.6	0.77–2.44	-	n.d.	n.d.	-	n.d.	n.d.	26	1.36 ± 0.6	0.77–2.44
PAT ^d^	70	17.81 ± 3	14.73–24.66	20	25.79 ± 5	21.69–29.98	30	35.86 ± 7	26.85–41.93	40	26.48 ± 9	14.73–41.93
ZON ^d^	-	n.d.	n.d.	30	11.53 ± 13	2.53–26.41	-	n.d.	n.d.	10	11.53 ± 13	2.53–26.41

^a^ Cider = cider with alcohol; ^b^ A.F. cider = alcohol free cider; ^c^ mycotoxin determined by LC-MS/MS; ^d^ mycotoxin determined by GC-MS/MS; ^e^ incidence (%): (number positive samples/number total samples) × 100.

**Table 5:** Co-occurrence mycotoxin data on the sum of the concentrations found in the same sample and combined risk characterization from different beverages.

Co-Occurrence	Sample (N)	Sum. C.Min.	Sum. C.Max.	Sum PDI Min.	TDI	Sum PDI Max.	TDI
		(µg/L)	(µg/L)	(ng/kg bw/day)	(%)	(ng/kg bw/day)	(%)
Two mycotoxins							
AOH, AFB_1_	Beer (1)	22.95	-	13.11	-	-	-
NIV, AOH	Beer (1)	22.84	-	13.05	0.66	-	-
DON, OTA	Beer with lemonade (1)	10.86	-	1.24	0.26	-	-
AOH, AME	A.F. Wine (1)	16.56	-	0.79		-	-
OTA, AOH	A.F. Wine (2)	4.45	5.78	0.19	0.14	0.24	0.28
OTA, NIV	Wine with lemonade (1)	12.22	-	0.52	0.30	-	-
OTA, β-ZAL	Wine with lemonade (1)	30.19	-	1.29	0.93	-	-
PAT, OTA	Cava (6)	17.60	25.52	0.25	0.18	0.36	0.18
ZON, βZAL	Cider (2)	50.90	129.37	0.41	0.17	1.10	0.44
PAT, βZAL	Cider (2)	46.86	63.28	0.40	0.13	0.54	0.17
Three mycotoxins							
DON, AOH, AFB_1_	Beer (2)	30.22	46.30	17.26	0.49	26.45	0.50
HT-2, AOH, AFB_1_	Beer (1)	67.29	-	38.45	10.62	-	-
AOH, AFB_1_, AFG_1_	Beer (1)	17.80	-	10.17	-	-	-
βZAL, AOH, OTA	Beer with lemonade (1)	52.05	-	5.94	4.09	-	-
DON, AOH, OTA	Beer with lemonade (2)	11.05	16.56	1.26	0.26	1.89	0.27
DON, OTA, AME	Wine (1)	11.98	-	1.37	1.04	-	-
DON, 15ADON, NEO	Wine (1)	35.64	-	4.07	0.24	-	-
DON, NEO, PAT	Wine (1)	59.51	-	6.80	1.15	-	-
DON, PAT, AME	Wine (1)	103.92	-	11.87	2.61	-	-
PAT, AOH, AME	A.F. Wine (1)	39.17	-	1.67	0.16	-	-
OTA, AOH, AME	A.F. Wine (2)	16.08	28.43	0.68	0.15	1.21	0.37
PAT, AOH, OTA	A.F. Wine (1)	17.57	-	0.75	0.51	-	-
PAT, OTA, AME	A.F. Wine (1)	47.37	-	2.03	-	0.36	-
Four mycotoxins						-	
DON, NEO, AOH, AFB_1_	Beer (4)	39.54	48.32	22.59	0.49	27.61	0.50
DON, HT-2, AOH, AFB_1_	Beer (2)	52.44	54.96	29.96	9.09	31.40	10.80
NIV, βZAL, AOH, OTA	Beer with lemonade (1)	60.46	-	6.90	3.55	-	-
DON, 15ADON, βZAL, AOH	A.F. Beer (1)	77.52	-	8.85	1.69	-	-
DON, βZAL, AOH, AFG_1_	A.F. Beer (2)	68.86	79.44	7.84	1.76	9.07	2.34
DON, 15ADON, NEO, PAT	Wine (1)	51.15	-	5.84	0.70	-	-
PAT, OTA, AOH, AME	Wine (2)	44.37	77.93	5.07	1.01	8.91	1.42
NIV, OTA, AOH, AME	Wine (1)	42.07	-	4.81	1.06	-	-
Five mycotoxins							
DON, HT2, NIV, AOH, AFB_1_	Beer (1)	83.20	-	47.54	9.15	-	-
DON, NIV, AOH, AFB_1_, AFG_1_	Beer (1)	44.73	-	25.56	0.93	-	-
DON, NIV, NEO, AOH, AFB_1_	Beer (1)	60.37	-	31.21	0.48	-	-
DON, HT-2, AOH, AFB_1_, AFG_1_	Beer (2)	52.97	56.15	30.26	10.02	32.08	15.21
DON, HT-2, AOH, AFB_1_, AFG_1_	A.F. Beer (1)	67.17	-	7.67	1.38	-	-
DON, NIV, AOH, AFB_1_, AFG_1_	A.F. Beer (1)	53.84	-	6.15	0.10	-	-
DON, 15ADON, βZAL, AOH, AFG_1_	A.F. Beer (1)	91.48	-	10.45	2.29	-	-
DON, HT2, βZAL, AOH, OTA	Beer with lemonade (1)	72.46	-	8.28	5.20	-	-
HT-2, NIV, PAT, AOH, AME	Wine (1)	63.14	-	5.41	0.88	-	-
DON, NEO, OTA, AOH, AME	Wine (1)	36.90	-	4.22	1.64	-	-
DON, 15ADON, HT-2, NEO, PAT	Wine (1)	80.63	-	9.21	0.94	-	-
DON, 15ADON, NEO, AOH, AME	Wine (1)	61.65	-	7.04	0.22	-	-
HT2, NIV, PAT, AOH, AME	Wine (1)	63.14	-	5.41	0.88	-	-
Six mycotoxins							-
DON, NIV, NEO, AOH, AFB_1_, AFG_1_	Beer (1)	60.37	-	34.49	0.49	-	-
DON, 15ADON, βZAL, AOH, AFB_1_, AFG_1_	A.F. Beer (1)	104.82	-	11.97	2.78	-	-
DON, NIV, PAT, AOH, AFB_1_, AFG_1_	A.F. Beer (1)	84.56	-	9.66	1.43	-	-
DON, HT-2, NIV, βZAL, AOH, OTA	Beer with lemonade (1)	83.15	-	8.53	6.14	-	-
DON, NIV, ZON, βZAL, AOH, OTA	Beer with lemonade(1)	77.89	-	8.90	3.55	-	-
DON, NEO, PAT, OTA, AOH, AME	Wine (1)	51.15	-	5.85	0.79	-	-
DON, 15ADON, NIV, NEO, PAT, AOH	Wine (1)	80.63	-	9.21	0.94	-	-
Eight mycotoxins						-	-
DON, 15ADON, NIV, NEO, ZON, AOH, AFB_1_, AFG_1_	A.F. Beer (1)	108.10	-	12.35	0.94	-	-
Ten mycotoxins						-	-
DON, T-2, HT-2, NIV, NEO, PAT, ZON, AOH, AFB_1_, AFG_1_	AF. Beer (1)	185.15	-	21.16	7.62	-	-

**Table 6:** Mycotoxin exposure calculated for adult population through alcoholic and non-alcoholic beverage consumption.

	Beer		A.F. Beer		Beer with Lemonade		Wine		A.F. Wine		Wine with Lemonade		Cava		Cider Alcohol		A.F. Cider	
Mycotoxin	PDI (ng/kg bw/day)	% TDI	PDI (ng/kg bw/day)	% TDI	PDI (ng/kg bw/day)	% TDI	PDI (ng/kg bw/day)	% TDI	PDI (ng/kg bw day)	% TDI	PDI (ng/kg bw/day)	% TDI	PDI (ng kg bw/day)	% TDI	PDI (ng/kg bw/day)	% TDI	PDI (ng/kg bw/day)	% TDI
AFB1	0.63	-	0.32	-	-	-	-	-	-		-	-	-	-	-	-	-	-
AFG1	0.88	-	0.10	-	-	-	-	-	-	-	-	-	-	-	-	-	-	-
AME	-	-	-	-	-	-	0.89	-	0.80	-	0.60	-	-	-	-	-	-	-
AOH	15.01	-	3.5	-	0.54	-	0.92	-	0.26	-	0.12	-	0.5	-	-	-	-	-
15ADON	-	-	1.47	0.14	-	-	1.33	0.13	-	-	-	-	-	-	-	-	-	-
β-ZAL	-	-	5.25	2.10	5.23	2.09	-	-	-	-	1.26	0.50	-	-	0.60	0.24	-	-
DON	5.21	0.52	1.17	0.11	1.06	0.10	1.04	0.10	-	-	-	-	-	-	-	-	-	-
HT-2	9.82	9.82	1.76	1.76	1.84	1.84	1.84	1.84	-	-	-	-	-	-	-	-	-	-
NEO	8.54	-	1.84	-	-	-	1.69	-	-	-	-	-	-	-	-	-	-	-
NIV	6.24	0.52	1.25	0.10	1.13	0.09	2.51	0.20	-	-	0.51	0.04	-	-	-	-	-	-
OTA	-	-	-	-	0.22	1.31	0.13	0.77	0.05	0.31	0.06	0.35	0.03	0.18	-	-	-	-
PAT	-	-	5.25	1.31	-	-	3.74	0.93	0.86	0.21	-	-	0.40	0.10	0.25	0.06	0.35	0.08
T-2	-	-	3.64	3.64	-	-	-		-	-	-	-	-	-	-	-	-	-
ZON	-	-	1.81	0.72	1.66	0.66	-	-	-	-	-	-	-	-	0.11	0.04	-	-

PDI: probable daily intake; TDI: tolerable daily intake.
